# p53 immunohistochemistry in endometrial cancer: clinical and molecular correlates in the PORTEC-3 trial

**DOI:** 10.1038/s41379-022-01102-x

**Published:** 2022-06-25

**Authors:** Lisa Vermij, Alicia Léon-Castillo, Naveena Singh, Melanie E. Powell, Richard J. Edmondson, Catherine Genestie, Pearly Khaw, Jan Pyman, C. Meg McLachlin, Prafull Ghatage, Stephanie M. de Boer, Hans W. Nijman, Vincent T. H. B. M. Smit, Emma J. Crosbie, Alexandra Leary, Carien L. Creutzberg, Nanda Horeweg, Tjalling Bosse, for the TransPORTEC consortium, N. Horeweg, S. M. de Boer, C. L. Creutzberg, T. Bosse, V. T. H. B. M. Smit, J. Kroep, R. A. Nout, H. W. Nijman, M. de Bruyn, M. E. Powell, N. Singh, H. C. Kitchener, E. Crosbie, R. Edmondson, D. N. Church, A. Leary, L. Mileshkin, P. M. Pollock, H. MacKay

**Affiliations:** 1 Departments of Pathology, Leiden University Medical Center, Leiden, The Netherlands; 2 Departments of Pathology, Barts Health NHS Trust, London, UK; 3 Clinical Oncology, Barts Health NHS Trust, London, UK; 4 Division of Cancer Sciences, University of Manchester, St Mary’s Hospital, Manchester, UK; 5 Departments of Pathology, Gustave Roussy, Villejuif, France; 6 Division of Radiation Oncology, Peter MacCallum Cancer Centre, Melbourne, VIC Australia; 7 Department of Anatomical Pathology, Royal Women’s Hospital, Parkville, VIC Australia; 8 Department of Pathology and Laboratory Medicine, Western University, London, ON Canada; 9 Department of Gynecological Oncology, Tom Baker Cancer Centre, Calgary, AB Canada; 10 Radiation Oncology, Leiden University Medical Center, Leiden, The Netherlands; 11 Department of Gynecology, University Medical Center Groningen, University of Groningen, Groningen, The Netherlands; 12 Department of Obstetrics and Gynaecology, Manchester University NHS Foundation Trust, Manchester Academic Health Science Centre, Manchester, UK; 13 Medical Oncology, Gustave Roussy, Villejuif, France; 14 Department of Radiation Oncology, Leiden University Medical Center, Leiden, The Netherlands; 15 Department of Pathology, Leiden University Medical Center, Leiden, The Netherlands; 16 Department of Medical Oncology, Leiden University Medical Center, Leiden, The Netherlands; 17 Department of Radiation Oncology, Erasmus MC Cancer Center, Rotterdam, The Netherlands; 18 Department of Gynaecologic Oncology, University Medical Center Groningen, Groningen, The Netherlands; 19 Department of Clinical Oncology, Barts Health NHS Trust, London, UK; 20 Department of Cellular Pathology, Barts Health NHS Trust, London, UK; 21 Manchester Academic Health Science Centre, St Mary’s Hospital, Obstetrics and Gynaecology, Manchester, UK; 22 Oxford University Hospitals NHS Foundation Trust, Oxford NIHR Comprehensive Biomedical Research Centre, Oxford, UK; 23 Department of Radio-therapy, Institut Gustave Roussy, Villejuif, France; 24 Division of Cancer Medicine, Peter MacCallum Cancer Centre, Melbourne, VIC Australia; 25 School of Biomedical Sciences, Faculty of Health, Queensland University of Technology, Translational Research Institute, Princess Alexandra Hospital Campus, Brisbane, QLD Australia; 26 Odette Cancer Center, Sunnybrook Health Sciences Centre, Medical Oncology, Toronto, ON Canada

**Keywords:** Endometrial cancer, Prognostic markers

## Abstract

Standard molecular classification of endometrial cancers (EC) is now endorsed by the WHO and identifies p53-abnormal (p53abn) EC as the subgroup with the poorest prognosis and the most likely to benefit from adjuvant chemo(radio)therapy. P53abn EC are *POLE* wildtype, mismatch repair proficient and show abnormal immunohistochemical (IHC) staining for p53. Correct interpretation of routinely performed p53 IHC has therefore become of paramount importance. We aimed to comprehensively investigate abnormal p53 IHC patterns and their relation to clinicopathological and molecular features. Tumor material of 411 molecularly classified high-risk EC from consenting patients from the PORTEC-3 clinical trial were collected. p53 IHC was successful in 408 EC and was considered abnormal when the tumor showed a mutant expression pattern (including subclonal): overexpression, null or cytoplasmic. The presence of pathogenic mutations was determined by next generation sequencing (NGS). Abnormal p53 expression was observed in 131/408 (32%) tumors. The most common abnormal p53 IHC pattern was overexpression (*n* = 89, 68%), followed by null (*n* = 12, 9%) and cytoplasmic (*n* = 3, 2%). Subclonal abnormal p53 staining was observed in 27 cases (21%), which was frequently but not exclusively, associated with *POLE* mutations and/or MMRd (*n* = 22/27; *p* < 0.001). Agreement between p53 IHC and *TP53* NGS was observed in 90.7%, resulting in a sensitivity and specificity of 83.6% and 94.3%, respectively. Excluding *POLE*mut and MMRd EC, as per the WHO-endorsed algorithm, increased the accuracy to 94.5% with sensitivity and specificity of 95.0% and 94.1%, respectively. Our data shows that awareness of the abnormal p53 IHC patterns are prerequisites for correct EC molecular classification. Subclonal abnormal p53 expression is a strong indicator for *POLE*mut and/or MMRd EC. No significant differences in clinical outcomes were observed among the abnormal p53 IHC patterns. Our data support use of the WHO-endorsed algorithm and combining the different abnormal p53 IHC patterns into one diagnostic entity (p53abn EC).

## Introduction

In 2020, the World Health Organization (WHO) endorsed the molecular classification of endometrial carcinomas (EC) using surrogate markers and following a stepwise diagnostic algorithm^1,2^. This approach categorizes EC into four molecular classes with validated and reproducible prognostic significance^3–6^. Following this algorithm, all EC harboring a pathogenic mutation in the exonuclease domain of DNA polymerase epsilon (*POLE)* are classified as *POLE*-ultramutated (*POLE*mut) EC, regardless of mismatch repair (MMR) or p53 immunohistochemical (IHC) status. *POLE* wildtype (*POLE*wt) EC with loss of expression in one of the MMR proteins are classified as MMR-deficient (MMRd) EC, independent of p53 IHC status. The final step in the diagnostic algorithm uses p53 IHC to distinguish between EC without a specific molecular profile (NSMP) and p53-abnormal (p53abn) EC.

Most studies on the prognostic performance of this diagnostic algorithm have utilized p53 IHC to distinguish between p53abn and NSMP EC. The use of p53 IHC in the diagnostic algorithm is convenient, as it is widely available, inexpensive and interpretable by a (gyneco)pathologist. Some practices, however, use *TP53* sequencing methods to molecularly classify EC as p53abn. Recent studies showed that abnormal p53 IHC reliably identifies cases with *TP53* mutation in ovarian carcinoma (100% specificity and 96% sensitivity) and in EC biopsies (94% specificity and 91% sensitivity)^7,8^. This high conformity is reached when the three different abnormal p53 IHC patterns are recognized. The commonest of these is ‘mutant overexpression’ in which the majority (80–100%) of tumor cells show strong nuclear expression of p53^9^. This mutant overexpression p53 IHC pattern is most often associated with missense mutations in the DNA binding domain of *TP53*. Other tumors show complete absence of p53 expression (‘null mutant’ pattern) and frequently have frameshift or nonsense mutations encoding truncated p53 protein. More recently, a third abnormal p53 IHC pattern has been recognized: overexpression of p53 in the cytoplasm of the tumor cells caused by mutations in the tetramerization or C-terminal domain of *TP53*^7^. Given the reported high concordance between p53 IHC and sequencing results, it is hypothesized that replacing p53 IHC with next generation sequencing (NGS) with a panel that covers the complete *TP53* gene would give comparable results, and that these two techniques (IHC or NGS) may be used interchangeably.

Recent reports have indicated that abnormal p53 expression can also occur confined to a distinct geographic area of the tumor, a pattern that has been termed ‘subclonal’ abnormal p53 expression^8,10^. In these cases, most typically, a well-defined area within a tumor shows an abnormal p53 IHC pattern, whilst the remaining tumor shows wildtype p53 expression. Depending on the area of the tumor from which DNA is extracted, it is conceivable that in tumors with subclonal abnormal p53 expression, a *TP53* mutation is not always identified by sequencing methods. Moreover, subclonal abnormal p53 expression in EC is still relatively understudied and its definition is poorly described. Some have suggested the use of a threshold of at least 10% abnormal p53 expression to define subclonality^4^. However, in our practice and clinical trials we have also observed unequivocal mutant overexpression of p53 in tiny foci (<10%) within an otherwise p53 wildtype tumor.

The correct interpretation of p53 IHC is essential for the molecular EC classification diagnostic algorithm, because it significantly impacts a patient’s individual risk assessment and subsequent treatment^11^. Most studies validating the molecular EC classification have reported considerable differences in clinical outcomes between patients with NSMP and p53abn EC, with the latter group persistently showing poor clinical outcomes^3–6^. Furthermore, recent data suggest that patients with p53abn high-risk EC benefit from the addition of chemotherapy to adjuvant external beam radiotherapy in the adjuvant treatment setting^4^. This observation has led to the recommendation of combined adjuvant radiotherapy with chemotherapy for patients with stage I (with myometrial invasion)-IVA p53abn EC in the recently updated ESGO-ESTRO-ESP EC guideline^11^. Furthermore, therapeutic agents such as poly-(ADP-ribose) polymerase (PARP) inhibitors in homologous deficient (HRD) EC and anti-HER2 therapies in HER2-positive EC are currently being explored^12–14^. Both HRD and HER2-positivity are highly, and in some studies exclusively, associated with p53abn EC, supporting p53abn subgroup-specific testing for HRD and HER2 status^15,16^.

In the current study, we aimed to comprehensively describe the clinicopathological and molecular features of p53abn EC, and to evaluate the concordance between p53 IHC and *TP53* sequencing, in a large series of hysterectomy-derived EC samples from patients who participated in the PORTEC-3 clinical trial.

## Methods

### Patient and tissue selection

The study population comprised 424 consenting patients included in the international PORTEC-3 clinical trial and tumor material collected by the *Trans*PORTEC research consortium. The design and results of the trial have been published previously^4,17^. In brief, 660 eligible patients with high-risk EC were randomly assigned 1:1 to postoperative chemoradiotherapy (CTRT) versus radiotherapy (RT) alone. Pathological inclusion criteria for the PORTEC-3 trial were: International Federation of Gynaecology and Obstetrics (FIGO) 2009 stage IA grade 3 endometrioid EC (EEC) with LVSI; stage IB grade 3 EEC; stage II-IIIC EEC of any grade; or non-endometrioid EC with stages IA (with invasion), IB-IIIC. Patients with uterine (carcino)sarcomas were excluded from participation in the trial. Eligibility was confirmed by upfront central pathology review by reference gynaecopathologists. The study was approved by the ethics committees at all participating centers.

Molecular subgroup assignment (*POLE*mut, MMRd, NSMP or p53abn) according to the WHO 2020 guidelines was successful in 411 EC for whom tumor blocks were available. In our previous study^4^, a threshold of more than 10% for subclonal abnormal p53 expression was used to assign a MMR proficient and *POLE* wildtype tumor to the p53abn subgroup. Patient, tumor and treatment characteristics did not differ significantly between PORTEC-3 trial patients included and excluded from the molecular analyses, as reported previously^4^.

### Immunohistochemistry

**Fig. 1:**
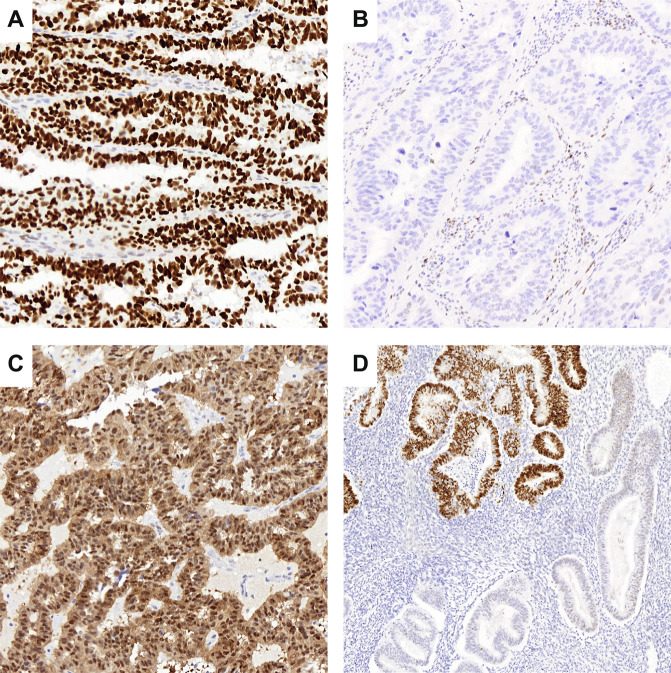
Representative examples of abnormal p53 immunohistochemical staining patterns. **A** Mutant overexpression, **B** null mutant, **C** cytoplasmic, and **D** subclonal abnormal p53 expression.

For each case, one representative formalin-fixed paraffin-embedded (FFPE) tumor block had been previously selected by a pathologist during central pathology review of the PORTEC-3 trial. These blocks were stored for translational research at the department of pathology of the Leiden University Medical. Immunohistochemical staining of p53 (DO-7, 1:200; Agilent DAKO) was retrospectively performed on 4 μm whole slides in batches of 50–100 slides as described previously^4^. All p53 IHC slides had been initially scored by two pathologists for the purpose of the molecular classification^4^. This previous scoring did not differentiate any of the known p53 mutational patterns, nor was the percentage of mutant-type staining noted. Therefore, for the purpose of the current study we re-evaluated the p53-stained slides blinded to previous scores and any of the molecular data using a more detailed scoring system. Re-evaluation of all p53 IHC slides was done by one of the two original pathologists (ALC) and discordant scores were discussed at a consensus meeting with both pathologists (ALC and TB). The interval between the first and second evaluation of p53 IHC was 1.5 years. For this re-evaluation, abnormal p53 expression was categorized as follows; strong positive p53 expression in >80% of the tumor nuclei (mutant overexpression), complete absence of p53 expression with a positive internal control (null mutant) or significant cytoplasmic p53 expression (cytoplasmic) in >80% of the tumor^8^. Subclonal abnormal p53 expression was defined as any abrupt and regional abnormal p53 expression in less than 80% of the tumor volume. The percentage of subclonality was noted. Representative examples of all abnormal p53 IHC patterns are shown in Fig. 1. Wildtype p53 expression was defined as nuclear staining of variable intensity in 1–80% of the tumor^8^. P53 IHC was considered failed when there was complete absence of p53 expression without an internal positive control, defined as scattered positive staining of p53 with variable intensity in fibroblasts, endothelial cells or lymphocytes^9^. In addition, for the purpose of this study, “ambiguous” p53 expression was not considered a category, so that all cases got assigned one of the five p53 IHC patterns. This resulted in re-assignment of nine cases originally scored as ambiguous into p53 wildtype or p53-abnormal. Twelve cases, originally scored as p53 wildtype, upon re-evaluation were scored “any subclonal abnormal p53” resulting in a re-assignment to p53-abnormal molecular class. In addition, two cases originally scored as wildtype were re-assigned as p53-abnormal. The p53 IHC scores obtained after re-examination of the p53 IHC slides were used to evaluate the agreement with *TP53* mutation status.

### Next-generation sequencing

Targeted next-generation sequencing (NGS) had previously been performed on PORTEC-3 tumor samples. A detailed description of DNA isolation and sequencing was described previously^4^. In brief, tumor DNA was collected by taking three random 0.8 mm tumoral tissue cores. For samples with low tumor volume, DNA was enriched by microdissection of selected tumoral areas using 5–10 tissue slides. Although DNA was isolated from the same tumor block, the p53 IHC slide was not used to direct the area of microdissection. Samples were sequenced in batches of 48 samples using the AmpliSeq Cancer Hotspot Panel version 5, which included frequently mutated genes in EC and covered the *TP53* gene in its entirety. The presence of pathogenic mutations was assessed blinded from the p53 IHC scores. Only variants with a predefined minimum coverage of 100 reads and variant allele frequency (VAF) of 10% were considered. Pathogenicity of non-synonymous mutations was assessed using the public International Agency for Research on Cancer (IARC) *TP53* database^18^ and ClinVar database^19^. Only mutations classified as (likely) pathogenic were included in the analyses.

### Statistical analysis

Statistical analyses were performed with SPSS (Statistical Package of Social Science) version 25 (IBM, Armonk, NY, USA). Five-year recurrence-free survival (RFS) was estimated using the Kaplan–Meier’s methodology and compared between groups using the log-rank test. The diagnostic performance of p53 IHC was determined by calculating the accuracy, sensitivity and specificity of p53 IHC compared to *TP53* NGS analysis. A two-sided *p*-value < 0.05 was considered statistically significant.

## Results

### Clinicopathological characteristics

**Fig. 2:**
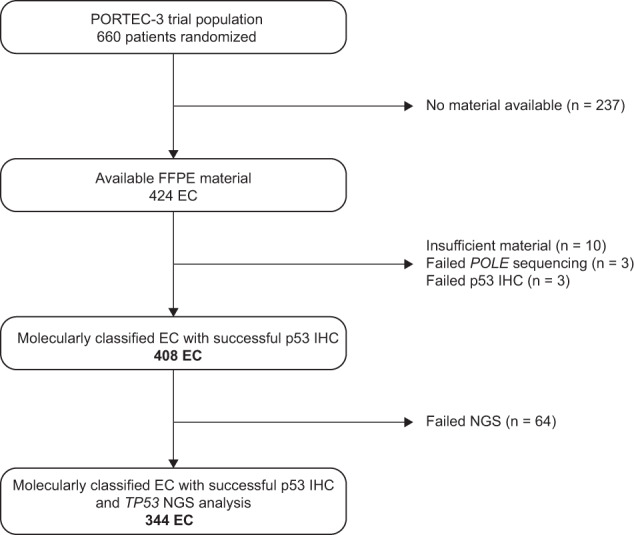
Flowchart of cohort selection. FFPE, formalin-fixed, paraffin-embedded; EC, endometrial cancer; IHC, immunohistochemistry; NGS, next generation sequencing.

p53 IHC was successful in 408 molecularly classified high-risk EC (Fig. 2). Of these, 344 (84.3%) cases also had successful *TP53* NGS analysis. Clinicopathological characteristics of the 408 cases included in this study are provided in Table 1. Most had endometrioid histology (*n* = 272, 66.7%) and FIGO stage II (*n* = 105, 25.7%) or stage III (*n* = 176, 43.1%) disease. We used hysterectomy samples in 96.6% (*n* = 394) of the cases.

**Table 1:** Patient and tumor characteristics.

	Total
	*n* = 408 (100%)
Age, years	
Mean (range)	61.2 (26.7–80.5)
Histotype	
Low grade endometrioid	160 (39.2)
High grade endometrioid	112 (27.5)
Serous	65 (15.9)
Clear cell	40 (9.8)
Mixed EEC-SEC	9 (2.2)
Mixed EEC-CCC	10 (2.5)
Other	12 (2.9)
Stage	
IA	54 (13.2)
IB	73 (17.9)
II	105 (25.7)
III	176 (43.1)
Specimen type	
Hysterectomy specimen	394 (96.6)
Curettage/biopsy	13 (3.2)
Lymph node	1 (0.2)

*EEC* endometrioid endometrial cancer, *SEC* serous endometrial cancer, *CCC* clear cell carcinoma.

### Evaluation of p53 IHC

Overall p53 IHC staining quality was of sufficient standard to allow interpretation as wild-type or abnormal; although the blocks were from many different hospitals across the world, the variability in fixation did not hamper assessment in the majority of cases. Wildtype p53 expression was observed in 277/408 (77.9%) cases. Abnormal p53 expression was observed in 131/408 (32.1%) of the cases. The most common abnormal p53 IHC pattern was mutant overexpression (*n* = 89/131, 67.9%). Complete absence of p53 expression (null mutant pattern) was observed in twelve (9.2%) cases and significant cytoplasmic staining in three (2.3%) cases. Subclonal abnormal p53 expression was observed in 27 cases (20.6%), at levels ranging from <10 to 75%. In all cases the subclonal p53 staining presented as mutant overexpression. A detailed description of all cases with subclonal abnormal p53 expression is provided in Table 2. Interestingly, seventeen cases showed the presence of (multifocal) subclonal foci with nuclear overexpression of p53, comprising far less than 10% of the tumor (Supplementary Fig. S1).

**Table 2:** Detailed description of endometrial cancers with subclonal abnormal p53 expression.

Case nr.	Molecular subgroup*	Histotype and grade	% of abnormal p53 expression	DNA isolation in p53 abn area	*TP53* mutation detected	Type of mutation	VAF	cDNA change	Amino acid change
1	MMRd	G2 EEC	<10%	Unknown	Failed	–	–	–	–
2	NSMP	G3 EEC	<10%	Unknown	Failed	–	–	–	–
3	*POLE*mut	mixed EEC-CCC	<10%	Unknown	No	–	–	–	–
4	*POLE*mut	G3 EEC	<10%	No	No	–	–	–	–
5	*POLE*mut	other	<10%	No	No	–	–	–	–
6	MMRd	G3 EEC	<10%	No	No	–	–	–	–
7	MMRd	G3 EEC	<10%	Yes	No	–	–	–	–
8	MMRd	G2 EEC	<10%	No	No	–	–	–	–
9	NSMP	G1 EEC	<10%	Yes	No	–	–	–	–
10	*POLE*mut	G3 EEC	<10%	No	Yes	Missense	0.19	c.775 G > T	p.Asp259Tyr
11	*POLE*mut	G3 EEC	<10%	Unknown	Yes	Nonsense	0.63	c.637 C > T	p.Arg213*
12	*POLE*mut	G3 EEC	<10%	No	Yes	Missense	0.38	c.245 C > T	p.Pro82Leu
						Missense	0.22	c.817 C > T	p.Arg273Cys
						Nonsense	0.40	c.916 C > T	p.Arg306*
13	MMRd	G1 EEC	<10%	No	Yes	Missense	0.19	c.524 G > A	p.Arg175His
14	MMRd	G3 EEC	<10%	No	Yes	Missense	0.47	c.524 G > A	p.Arg175His
15	MMRd	G3 EEC	<10%	No	Yes	Missense	0.26	c.526 T > A	p.Cys176Ser
						Frameshift	0.14	c.287delC	p.Ser96LeufsTer27
16	MMRd	G2 EEC	<10%	No	Yes	Missense	0.27	c.374 C > T	p.Thr125Met
17	NSMP	G2 EEC	<10%	Unknown	Yes	Missense	0.33	c.743 G > A	p.Arg248Gln
18	*POLE*mut	G3 EEC	20%	No	Yes	Missense	0.51	c.827 C > T	p.Ala276Val
19	MMRd	G3 EEC	20%	Unknown	Yes	Missense	0.28	c.734 G > A	p.Gly245Asp
20	*POLE*mut	EEC-SEC	25%	No	Yes	Nonsense	0.16	c.637 C > T	p.Arg213*
21	MMRd	G3 EEC	30%	Unknown	Failed	–	–	–	–
22	MMRd	G3 EEC	40%	Yes	No	–	–	–	–
23	*POLE*mut	G3 EEC	60%	Unknown	Yes	Nonsense	0.19	c.916 C > T	p.Arg306*
24	MMRd	G3 EEC	60%	Yes	Yes	Missense	0.31	c.536 A > G	p.His179Arg
25	p53abn	other	60%	Unknown	Yes	Missense	0.53	c.537 T > G	p.His179Gln
26	MMRd	G3 EEC	70%	Yes	Yes	Missense	0.47	c.536 A > G	p.His179Arg
						Missense	0.23	c.745 A > T	p.Arg249Trp
27	p53abn	SEC	75%	Unknown	Yes	Missense	0.81	c.470 T > A	p.Val157Asp

*Classified as per Leon-Castillo et al., JCO 2020, considering a 10% threshold to assign a tumor as p53abn EC.

*POLEmut* POLE mutant, *MMRd* mismatch repair deficient, *NSMP* no specific molecular profile, *p53abn* p53-abnormal, *EEC* endometrioid endometrial cancer, CCC clear cell carcinoma, SEC serous endometrial cancer, VAF variant allele frequency.

### Agreement of p53 IHC with TP53 mutation status

Conformity between p53 IHC and *TP53* NGS analysis was observed in 312 of 344 cases using a binary classification (Table 3). The accuracy of the concordance between p53 IHC and *TP53* NGS analysis was 90.7% (95% CI 87.6–93.8%), with a sensitivity of 83.6% (95% CI 79.7–87.5%) and a specificity of 94.3% (95% CI 91.8–96.7%). Of the 32 cases with discordances between p53 IHC and *TP53* NGS analysis, 22 cases were either *POLE*mut or MMRd EC. Confining the analysis to *POLE*wt and MMRp EC, as per the WHO-endorsed algorithm, increased the accuracy to 94.5% (95% CI 89.4–99.6%) with a sensitivity of 95.0% (95% CI 90.2–99.8%) and a specificity of 94.1% (95% CI 88.9–99.3%) (Table 3).

**Table 3:** Agreement between p53 immunohistochemistry and *TP53* NGS analysis.

	(Likely) pathogenic mutation on *TP53* NGS analysis			
	All EC		*POLE* wildtype and MMR proficient EC	
p53 IHC	Absent	Present	Absent	Present
Wildtype	**215**	19	**96**	4
Abnormal	13	**97**	6	**76**
Total	228	116	102	80
Accuracy	90.7% (95% CI 87.6–93.8%)		94.5% (95% CI 89.4–99.6%)	
Sensitivity	83.6% (95% CI 79.7–87.5%)		95.0% (95% CI 90.2–99.8%)	
Specificity	94.3% (95% CI 91.8–96.7%)		94.1% (95% CI 88.9–99.3%)	

In bold, EC with concordant p53 IHC and sequencing for *TP53* mutations.

*IHC* immunohistochemistry, *NGS* next generation sequencing, *EC* endometrial cancer, *MMR* mismatch repair, *CI* confidence interval.

### Discordant cases

**Fig. 3:**
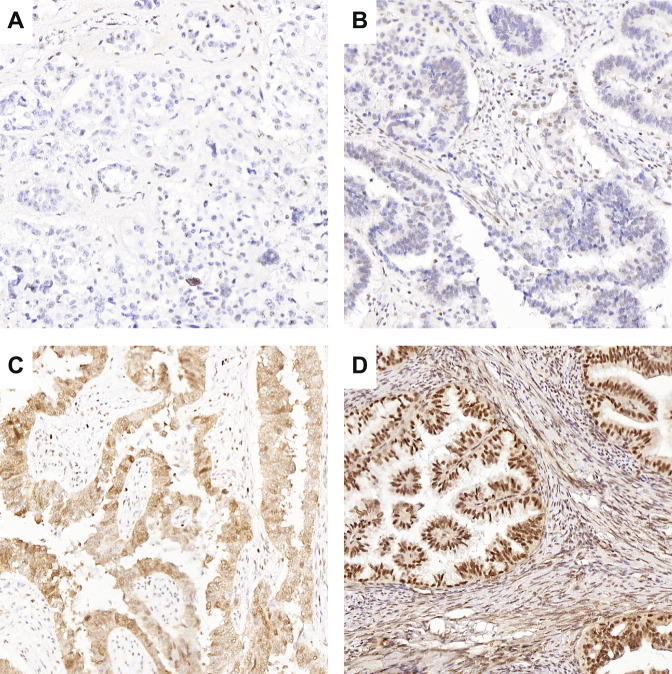
p53 immunohistochemical staining (IHC) of cases with discordant results between p53 IHC and *TP53* next generation sequencing (NGS), as a result of erroneously interpreted p53 IHC. Cases shown in (**A**–**C**) were scored p53 wildtype but did have a frameshift *TP53* mutation (**A**, **B**) and nonsense *TP53* mutation (**C**). In retrospect, p53 IHC should have been scored as null (**A**, **B**) and cytoplasmic (**C**). The case shown in (**D**) was assigned ‘p53 mutant overexpression’, however did not have a *TP53* mutation. Given the high expression in the stromal cells, this case was probably overstained.

In total, 32 cases (9.3%) had discordant results between p53 IHC and *TP53* NGS analysis (Supplementary Table S1). Firstly, there were 22 cases that were either *POLE*mut or MMRd EC. This included 5 *POLE*mut and 10 MMRd EC with a *TP53* mutation that did not result in abnormal p53 expression, and 3 *POLE*mut and 4 MMRd EC which showed subclonal abnormal p53 IHC with no detectable *TP53* mutation. In 4 cases the p53 immunostaining was likely erroneously interpreted. Two cases had missed null mutant patterns (Fig. 3A, B). In both cases a nonspecific nuclear blush was mistaken for wildtype p53 expression, which has been previously described as an artifact in true null mutant cases that can be encountered with more sensitive p53 IHC methods^9^. In addition, there was 1 case with a missed cytoplasmic p53 IHC pattern (Fig. 3C). In the fourth case the p53 IHC slide was overstained with nuclear positivity of the stroma and was therefore not reliably interpretable (Fig. 3D). Restaining p53 IHC in the latter case confirmed unequivocal wildtype p53 expression. The p53 IHC assignment of these 4 misinterpreted cases was corrected in the database for further analyses in this study (including the molecular landscape and survival analyses). The remaining 6 *POLE*wt and MMRp EC showed true discrepancies between IHC and NGS results: one case assigned as NSMP EC, based on wildtype p53 IHC, had a missense *TP53* mutation. Re-examination of the p53 IHC revealed unequivocal wildtype p53 staining. Four cases had the mutant overexpression p53 IHC pattern without a detected *TP53* mutation (Supplementary Fig. S2). All of these cases showed unambiguous mutant-type overexpression with optimal staining. Finally, 1 case with subclonal p53 IHC expression did not show a *TP53* mutation.

### Molecular landscape of p53-abnormal and/or TP53-mutated HREC

**Fig. 4:**
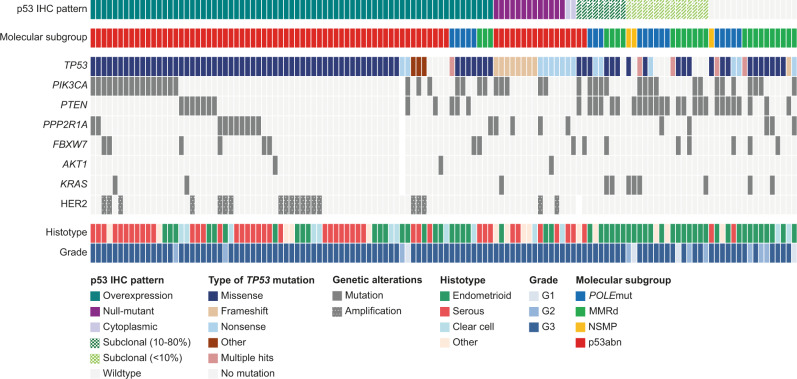
Histopathological and molecular characteristics of all high-risk endometrial cancers (*n* = 128) with abnormal p53 immunohistochemistry and/or a pathogenic *TP53* mutation. IHC immunohistochemistry, G grade, MMRd mismatch repair deficient, NSMP no specific molecular profile; p53abn, p53-abnormal.

Of the 344 cases with successful p53 IHC and *TP53* NGS analysis, 128 cases had abnormal p53 IHC expression and/or a pathogenic *TP53* mutation. The molecular landscape of these 128 cases is presented in Fig. 4. Within EC molecularly classified as p53abn, the most frequently observed genetic alterations were pathogenic mutations in *TP53* (*n* = 78/82, 95.1%), *PIK3CA* (*n* = 25/82, 30.5%), *PPP2R1A* (*n* = 14/82, 17.1%), *PTEN* (*n* = 10/82, 12.2%), and *FBXW7* (*n* = 8/82, 9.8%) and HER2 amplification (*n* = 20/82, 24.4%). Within the p53abn EC in our cohort, *PPP2R1A* mutations did not co-occur with *PTEN* and *KRAS* mutations, and rarely co-occurred with *FBXW7* mutations (*n* = 1/78). Half of the p53abn EC were of serous histologic subtype (*n* = 41, 50.0%), followed by endometrioid (*n* = 21, 25.6%) and clear cell (*n* = 10, 12.2%) histologic subtypes.

Mutant overexpression of p53 was the most prevalent abnormal p53 IHC pattern and was found in EC with missense *TP53* mutations (*n* = 63/73, 86.3%), nonsense mutations (*n* = 2/73, 2.7%), in-frame deletions (*n* = 2/73, 2.7%) and in one case (1.7%) with a splice-site mutation. Mutant overexpression of p53 was observed in five *POLE*mut and three MMRd EC. One of the *POLE*mut EC had two pathogenic *TP53* mutations (missense and nonsense). Eighteen cases with mutant overexpression of p53 were HER2-positive (*n* = 18/73, 24.7%), compared to two cases within the null mutant group (*n* = 2/13, 15.4%).

The null mutant p53 IHC pattern was associated with frameshift (*n* = 8/13, 61.5%) and nonsense (*n* = 5/13, 38.5%) *TP53* mutations. Two cases with cytoplasmic p53 expression showed a nonsense mutation in the tetramerization domain of p53. Neither null- or cytoplasmic p53 IHC patterns were encountered in the context of MMRd and *POLE*mut EC. Interestingly, none of the ECs with a null mutant or cytoplasmic p53 IHC pattern harbored *PTEN* mutations. In comparison, *PTEN* mutations were found in 13/74 (17.6%) EC with mutant overexpression of p53.

Cases with subclonal abnormal p53 expression showed a substantially different molecular profile, consistent with their molecular subgroup assignment as either MMRd or *POLE*mut EC. These cases had frequent mutations in *PTEN* (*n* = 18/24, 75.0%) and *PIK3CA* (*n* = 11/24, 45.8%) and were predominantly endometrioid EC (*n* = 19/24, 79.2%). In two thirds of the cases with a subclonal abnormal p53 IHC pattern (*n* = 16/24, 66.7%) a *TP53* mutation was identified, even in 8 out of 15 cases with abnormal p53 expression in <10% of the tumor. Of interest, subclonal abnormal p53 expression was observed in two cases with non-endometrioid histology that were *POLE*wt and MMRp. In both cases subclonal abnormal p53 expression was observed in >50% of the tumor and a *TP53* mutation was confirmed.

Like cases with a subclonal abnormal p53 IHC pattern, the majority of *TP53* mutant EC with wildtype p53 IHC were molecularly classified as *POLE*mut or MMRd (*n* = 15/16, 93.8%) and harbored frequent mutations in *PTEN* (*n* = 8/16, 50.0%) and *PIK3CA* (*n* = 7/16, 43.8%). There was one *POLE*wt and MMRp serous EC that showed wildtype p53 expression that did have a missense *TP53* mutation.

### Prognostic relevance of abnormal p53 IHC patterns

**Fig. 5:**
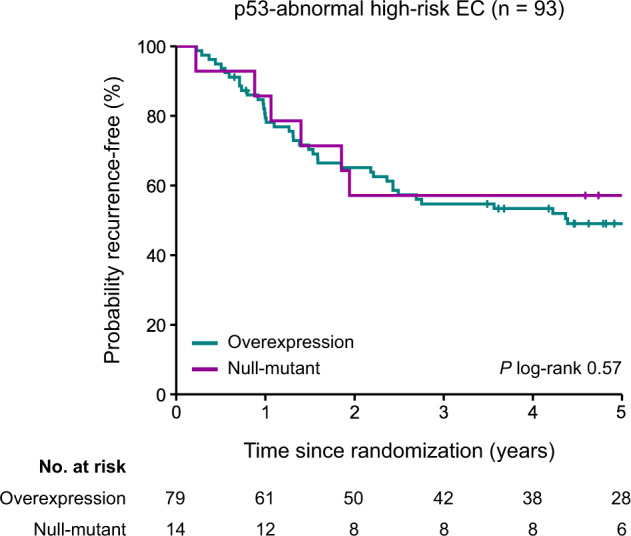
Kaplan–Meier curves for time to recurrence for patients with p53-abnormal high-risk endometrial cancers with mutant overexpression and null-mutant p53 immunohistochemical staining patterns (*n* = 93). EC endometrial cancer.

Finally, we investigated the prognostic relevance of the different abnormal p53 IHC patterns within the group of patients with EC assigned as p53abn. There were no significant differences in 5-year recurrence rates between the mutant overexpression and null mutant p53 IHC patterns (49.1% [95% CI 37.6–60.6%] versus 57.1% [95% CI 31.2–83.0%] respectively, *p* = 0.57; Fig. 5). Of the three patients with cytoplasmic and two patients with subclonal abnormal p53 expression (in 60% and 75% of the tumor), the tumor recurred in one patient in both groups, resulting in overlapping survival curves (Supplementary Fig. S3A). Likewise, no significant differences in 5-year recurrence rate were observed among the different types of *TP53* mutation in patients with p53abn EC (Supplementary Fig. S3B).

## Discussion

In this large study of high-risk EC samples from hysterectomy specimens of patients included in the PORTEC-3 trial, we corroborate the abnormal p53 IHC patterns previously described in ovarian cancer and endometrial cancer biopsy samples^7,8^. We describe a novel finding that subclonal abnormal p53 expression can be observed in small multifocal areas comprising less than 10% of the tumor volume. We show that concordance between p53 IHC and *TP53* mutational analysis using NGS in EC hysterectomy samples is good with an estimated accuracy of 90.7%. Furthermore, we find no significant differences among the abnormal p53 IHC patterns with regard to clinicopathological and molecular characteristics, nor in clinical outcomes, which supports combining the different abnormal p53 IHC patterns into one diagnostic entity (p53abn EC).

Few studies have reported on subclonal abnormal p53 IHC patterns. We observed subclonal abnormal p53 expression in up to 7% of cases, comparable to the prevalence of 5% in EC biopsies enriched for p53 mutant cases reported by Singh et al.^8^. In this study, we discovered that subclonality can also present as small multifocal areas in less than 10% of the tumor volume. In eight of fifteen cases with <10% subclonal abnormal p53 expression, a *TP53* mutation was identified, supporting the hypothesis that this pattern represents true mutant subclones. In agreement with previous studies, we observed a strong association between subclonal abnormal p53 expression and *POLE* mutations and MMRd. Here, the *TP53* mutation is likely acquired at a later stage of tumor progression and previous work suggest this does not influence clinical behavior^20^. It has been argued that subclonal abnormal p53 expression can be a selection criterion for *POLE* testing in EC with normal MMR IHC expression. Although there may be some enrichment for *POLE*mut EC using this approach, a large number of *POLE*mut EC would not be identified^20^. Despite the strong association between subclonal abnormal p53 expression and *POLE*mut and MMRd EC, we observed five cases with subclonal abnormal p53 expression in *POLE*wt and MMRp EC. This suggests that EC can also acquire *TP53* mutations during tumor progression outside the context of *POLE*mut and MMRd EC. It is currently unclear whether these subclones become more dominant during tumor progression and subsequently increase the patients’ risk for metastases or recurrence. As a result, there is no consensus definition for subclonal abnormal p53 expression in this specific context. Previous studies have used a lower-limit cut-off of more than 10% for molecular subgroup assignment of these rare cases^4,21,22^. Given the lack of robust clinical data to suggest otherwise, we endorse the continued use of this threshold for uniformity.

We report a high agreement between p53 IHC and *TP53* NGS analysis of 90.7% overall and 94.5% when used as part of the recommended algorithm, comparable to the agreement reported in ovarian cancer and EC biopsies^7,8^. Most discordances between p53 IHC and *TP53* NGS analysis occurred in *POLE*mut and MMRd EC. These discordances might be explained by *TP53* mutations that occur at a later stage of tumor progression, resulting in subclonal abnormal p53 expression. Here, *TP53* mutations can be missed when DNA is not isolated from the area of the tumor showing abnormal p53 expression. In contrast, by coring FFPE tumor blocks it is possible that *TP53* mutations in p53 mutant subclones, that lie deeper than the whole slide p53 IHC, are being detected. In addition to the discordances in *POLE*mut and MMRd EC, we observed ten discordant cases which were eventually classified as NSMP or p53abn EC, all of which are discussed in more detail in the results. One case with subclonal p53 expression in less than 10% of the tumor did not have a *TP53* mutation. Five cases with unequivocal wildtype or mutant overexpression p53 IHC pattern that did not correspond with *TP53* NGS analysis. Four other discordances could be explained by erroneously interpreted p53 IHC. Three cases were called p53 wildtype by IHC but did have a *TP53* mutation. On review, these cases represented null mutant and cytoplasmic p53 expression, well-known pitfalls in p53 IHC interpretation. One other discordant case was called p53 abnormal by IHC without a *TP53* mutation found by NGS. Here, the p53 IHC slide was overstained with nuclear positivity of the stroma and was therefore not reliably interpretable. These discordant cases illustrate the importance of a quality-assured p53 immunostaining procedure, good internal controls, optimal pre-processing of tumor tissue and awareness of all the different abnormal p53 IHC patterns when molecularly classifying EC. We did not add external positive controls to our slides as, in this research-setting, we had a sufficient number of p53 mutant cases in each IHC run. However, in a clinical setting a good external on-slide positive control (e.g. a low-expressor, like tonsil) is common practice and highly recommended to support correct interpretation of cases that fall into the zone of potential misclassification.

Currently, it is unknown which method of p53 testing should be preferred when it comes to identifying p53abn EC. In previous studies, p53 IHC was most often used and has therefore proven prognostic value^3–6^. Given the high concordance between p53 IHC and *TP53* sequencing methods, it is likely that identical results can be obtained using *TP53* NGS analysis. However, in many practices NGS is not (yet) incorporated in routine clinical practice, as it is costly and requires the expertise of molecular biologists and bioinformaticians to analyze the results. Conversely, p53 IHC is widely available, relatively easy to interpret, allows for quick turn-around and is currently considerably cheaper than *TP53* sequencing. Taken together, although p53 IHC and NGS can probably be used interchangeably, there are sufficient arguments to favor p53 IHC to assign p53abn EC.

In agreement with existing literature, we have shown that mutant overexpression of p53 in EC is predominantly associated with missense *TP53* mutations. In addition, the null mutant pattern is associated with frameshift and nonsense *TP53* mutations, and the cytoplasmic IHC pattern with nonsense mutations in the tetramerization domain of *TP53*. In our study, the null mutant and cytoplasmic p53 IHC patterns were not identified within *POLE*mut and MMRd EC and in this group we did not see pathogenic *PTEN* mutations. These findings are interesting and may point towards a different underlying biology but should still be interpreted with caution as the total number of cases is limited.

To date, no studies have investigated the prognostic relevance of the different abnormal p53 IHC patterns and/or types of *TP53* mutation within EC. A study investigating 141 head and neck squamous cell carcinomas reported worse clinical outcomes for patients with truncating *TP53* mutations compared to patients with wildtype *TP53*, whilst missense *TP53* mutations did not influence prognosis^23^. In breast cancer literature inconsistent results have been reported regarding the impact of *TP53* mutation types on clinical outcome^24–27^. In our study, we did not observe a significant difference in recurrence rate between the different abnormal p53 IHC patterns observed in EC, nor between *TP53* mutation types, within p53abn EC. This finding supports the practice of combining all abnormal p53 IHC patterns into one diagnostic entity (p53abn EC).

Although our data supports the use of p53 IHC to assign p53abn subtype in practice, we acknowledge that our results are obtained in a research setting potentially impacting generalizability. First, we performed p53 IHC and *TP53* NGS runs in batches and all in a single laboratory, not reflecting the real-world. We know that in practice interlaboratory differences in p53 IHC protocols exist which may impact accuracy, as also highlighted by the work of Köbel et al. in EC and OC^7^. We therefore used the recommended DAKO D07 antibody, which showed the best performance and a high interobserver agreement in previous work^7^. Second, experts with experience in p53 IHC scoring in EC were used as reference in our study. It has been established by Singh et al. that experts have a high agreement of p53 IHC in EC biopsy samples^8^. However, it will be important for less experienced pathologists to get acquainted with the abnormal p53 IHC patterns in EC that are highlighted in this manuscript. To support training, we encourage the use of the previous published tutorial on this topic (http://www.gpec.ubc.ca/p53). Finally, improper preprocessing of tumor tissue may negatively impact the immunohistochemical staining of p53 and subsequently cause difficulties in interpretation. It was reassuring that we did not encounter any major difficulties, despite the use of blocks with variable fixation collected from many different hospitals. The agreement between p53 IHC and *TP53* NGS in our cohort of EC hysterectomy samples was comparable to the agreement reported in EC biopsy samples, which are generally better fixed^8^. In practice, a pre-operative EC (biopsy) sample is often available and could be utilized for confirmatory p53 IHC staining in cases with poor fixation of the hysterectomy specimen.

This is, to our knowledge, the first study investigating abnormal p53 IHC patterns and their association with clinicopathological and molecular features in EC hysterectomy samples. In addition to the previously reported four abnormal p53 IHC patterns in EC, we discovered that subclonal abnormal p53 expression can also present as small multifocal foci in <10% of the tumor. Furthermore, we have shown that subclonal abnormal p53 expression frequently occurs in the context of *POLE*mut and MMRd EC. We conclude that p53 IHC and *TP53* NGS analysis have high concordance, with a diagnostic accuracy comparable to EC biopsy samples and ovarian cancer. Proper technical performance of p53 IHC and acquaintance by the pathologist with the different abnormal p53 IHC patterns are prerequisites for correct molecular subgroup assignment. We recommend, at least, the use of *TP53* NGS analysis in EC with ambiguous p53 expression, in MMR proficient and *POLE* wildtype EC. Our findings continue to support the use of p53 IHC as part of the diagnostic algorithm for the molecular classification of EC.

## Supplementary information

Supplementary Material

## Data Availability

The PORTEC-3 dataset analyzed during the current study is available from the corresponding author on reasonable request.
