# Supplementary material for: p53 immunohistochemistry in endometrial cancer: clinical and molecular correlates in the PORTEC-3 trial
Source: Mod Pathol. 2022 Jun 25;35(10):1475–83. doi: 10.1038/s41379-022-01102-x (PMC7613653; doi:10.1038/s41379-022-01102-x)
Supplement: Supplementary file 1 — Supplementary Material [file 41379_2022_1102_MOESM1_ESM.pdf]

Figure S1. Representative example of an endometrial cancer, classified as ‘no specific molecular profile’, showing multifocal subclonal abnormal p53 expression in small foci, comprising less than 10% of the tumoral volume. A missense *TP53* mutation was found with next generation sequencing using DNA obtained from randomly taken tumor cores.

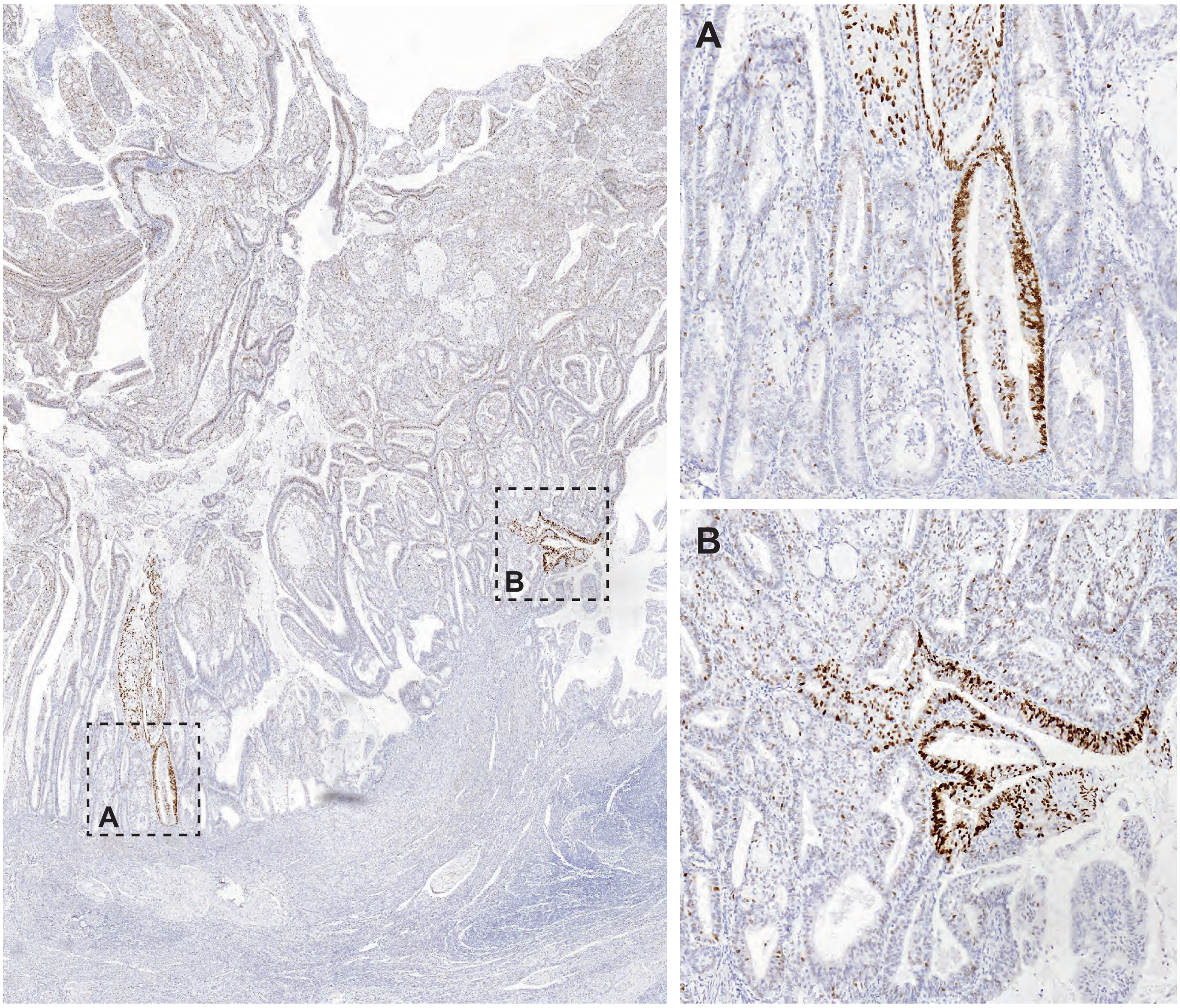

Figure S2. Cases with unequivocal mutant overexpression of p53 by immunohistochemistry (IHC) without evidence of a *TP53* mutation by next generation sequencing. For these cases, the assigned p53 status was not adjusted, given the very convincing IHC result.

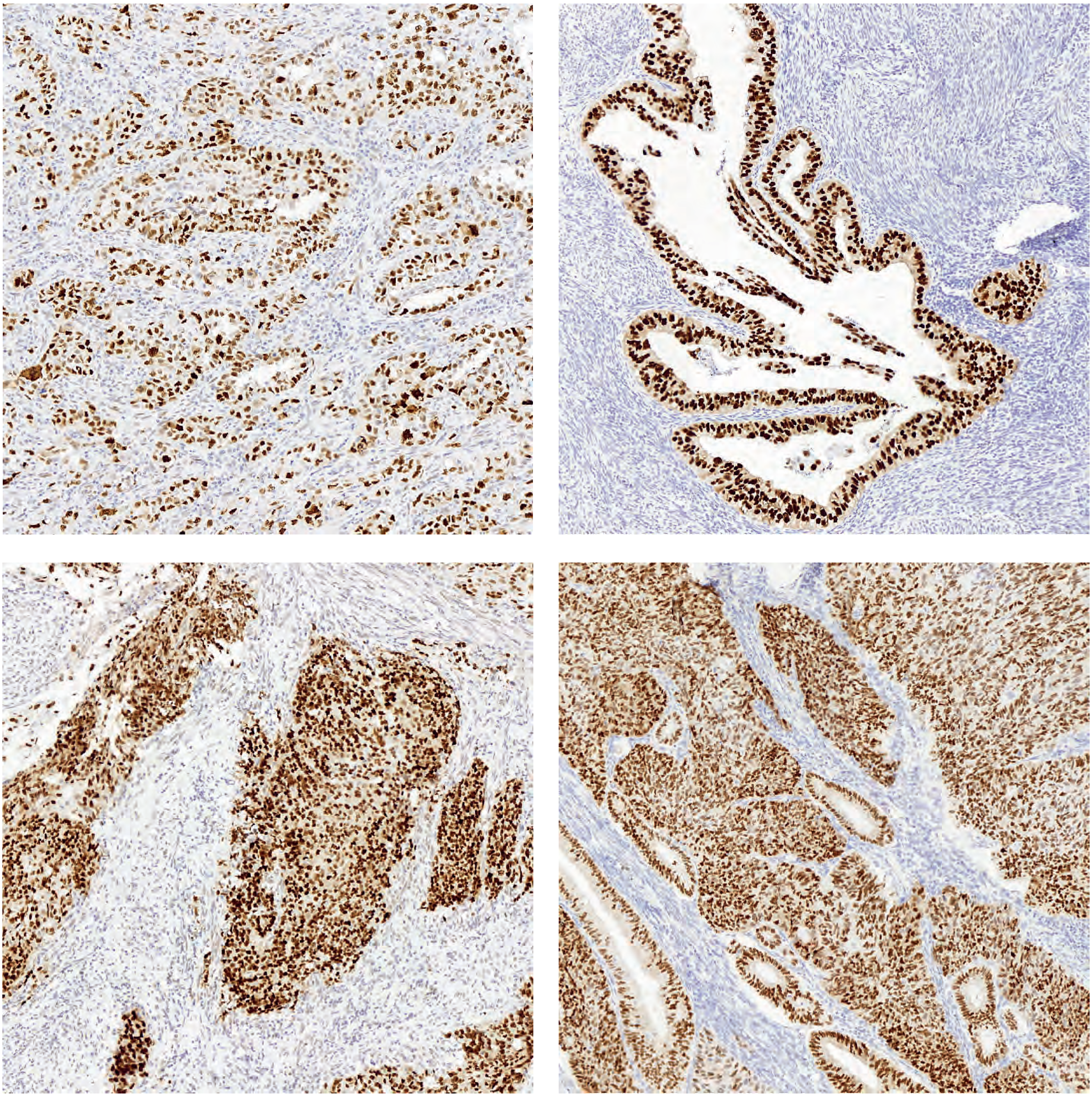

Figure S3. Time-to-recurrence for p53-abnormal high-risk endometrial cancers (n=98), stratified by p53 immunohistochemical staining pattern (A) and type of *TP53* mutation* (B).

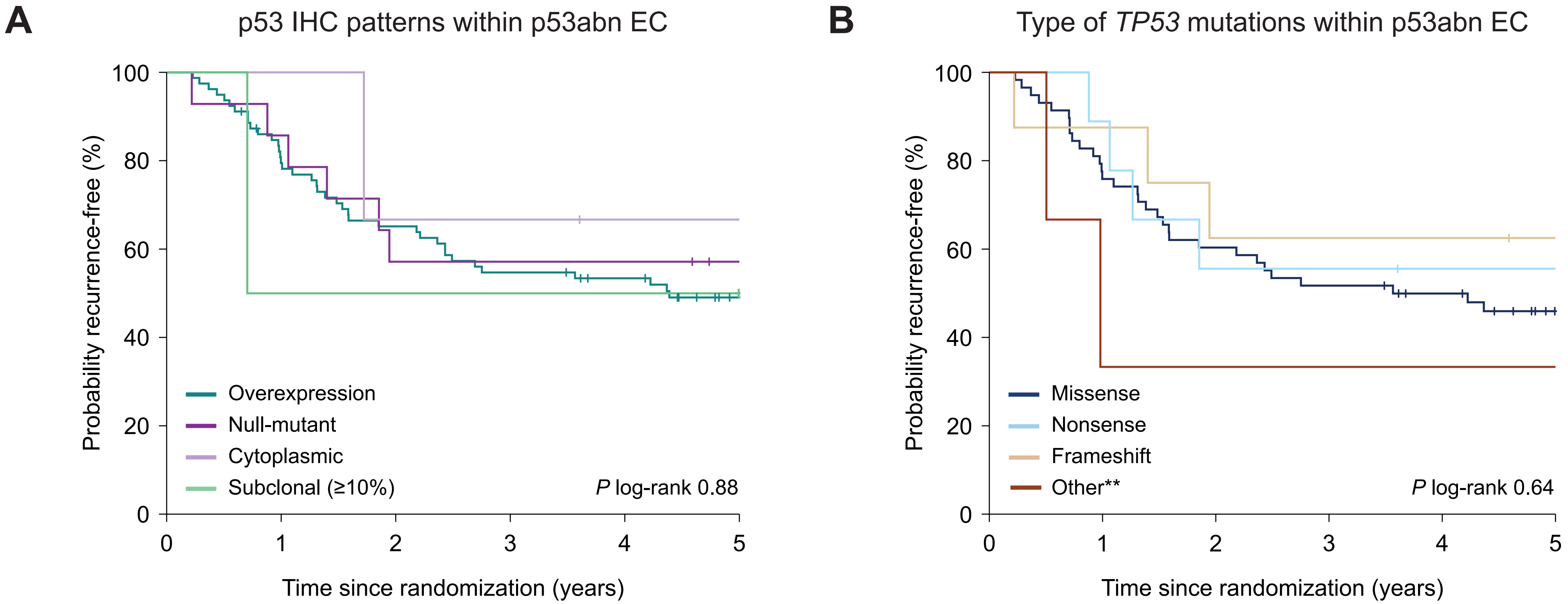

**No. at risk**	0	1	2	3	4	5
Overexpression	79	61	50	42	38	28
Null-mutant	14	12	8	8	8	6
Cytoplasmic	3	3	2	2	1	1
Subclonal (≥10%)	2	1	1	1	1	0

**No. at risk**	0	1	2	3	4	5
Missense	58	44	35	30	26	17
Nonsense	9	8	5	5	4	4
Frameshift	8	7	5	5	5	4
Other**	3	1	1	1	1	1

**p53-abnormal endometrial cancers without a TP53 mutation (n = 4) or with failed TP53 mutation analysis (n = 16) were excluded from the analysis*

*** Other mutations include two in-frame deletions and one splice-site TP53 mutation*

Table S1. Description of endometrial cancers with discordant results between p53 immunohistochemistry and TP53 NGS analysis.

Case number	Molecular subgroup*	Histotype and grade	p53 IHC pattern	*TP53* mutation detected	Type of mutation	VAF
1	POLE	GR3 EEC	Wildtype	Yes	Nonsense	0.47
2	POLE	GR3 EEC	Wildtype	Yes	Missense<br>Splice-site	0.28<br>0.27
3	POLE	GR3 EEC	Wildtype	Yes	Missense	0.24
4	POLE	Other	Wildtype	Yes	Missense	0.20
5	POLE	SEC	Wildtype	Yes	Nonsense	0.15
6	MMRd	GR1-2 EEC	Wildtype	Yes	Missense	0.30
7	MMRd	GR1-2 EEC	Wildtype	Yes	Missense	0.16
8	MMRd	GR1-2 EEC	Wildtype	Yes	Missense	0.25
9	MMRd	GR1-2 EEC	Wildtype	Yes	Nonsense	0.64
10	MMRd	GR3 EEC	Wildtype	Yes	Missense	0.21
11	MMRd	GR3 EEC	Wildtype	Yes	Missense	0.34
12	MMRd	GR3 EEC	Wildtype	Yes	Missense	0.26
13	MMRd	GR3 EEC	Wildtype	Yes	Missense<br>Nonsense	0.41<br>0.36
14	MMRd	CCC	Wildtype	Yes	Missense	0.20
15	MMRd	CCC	Wildtype	Yes	Frame-shift	0.73
16	POLE	GR3 EEC	Subclonal p53 expression (<10%)	No	-	
17	POLE	EEC-CCC	Subclonal p53 expression (<10%)	No	-	
18	POLE	Other	Subclonal p53 expression (<10%)	No	-	
19	MMRd	GR1-2 EEC	Subclonal p53 expression (<10%)	No	-	
20	NSMP	GR1-2 EEC	Subclonal p53 expression (<10%)	No	-	
21	MMRd	GR3 EEC	Subclonal p53 expression (<10%)	No	-	
22	MMRd	GR3 EEC	Subclonal p53 expression (≥10%)	No	-	
23	MMRd	GR3 EEC	Subclonal p53 expression (≥10%)	No	-	
24	NSMP	SEC	Wildtype	Yes	Frame-shift	0.63
25	NSMP	EEC-SEC	Wildtype	Yes	Frame-shift	0.88
26	NSMP	SEC	Wildtype	Yes	Nonsense	0.38
27	NSMP	GR1-2 EEC	Mutant overexpression	No	-	
28	NSMP	SEC	Wildtype	Yes	Missense	0.83
29	p53	CCC	Mutant overexpression	No	-	
30	p53	SEC	Mutant overexpression	No	-	
31	p53	GR3 EEC	Mutant overexpression	No	-	
32	p53	GR3 EEC	Mutant overexpression	No	-	

** Classified as per Leon-Castillo et al., JCO 2020, considering a 10% threshold to assign a tumor as p53abn EC.*

Abbreviations: POLEmut, POLE mutant; MMRd, mismatch repair deficient; NSMP, no specific molecular profile; p53abn, p53-abnormal; EEC, endometrioid endometrial cancer; CCC, clear cell carcinoma; SEC, serous endometrial cancer; VAF, variant allele frequency
